# A technique for non-deflating balloon catheter removal in female patients

**DOI:** 10.11604/pamj.2017.26.222.12291

**Published:** 2017-04-24

**Authors:** Eirini Giovannopoulou, Kostas Chondros

**Affiliations:** 1 University Hospital of Alexandroupolis, Democritus University of Thrace, Alexandroupolis, Greece; 2 Department of Urology, General Hospital of Rethymnon, Rethymnon, Greece

**Keywords:** Non-deflating balloon, catheter retention, catheter removal, balloon puncture

## Abstract

Removing a foley catheter can sometimes be a challenge, especially when it is related to non-deflating balloons which represent most of the cases. In female patients, due to their urethral anatomy, several techniques have been proposed for balloon puncture when other maneuvers have failed. We present a simple technique for non-deflating balloon catheter removal in an old female patient with a permanent indwelling foley catheter which required no anesthesia. The balloon was effectively punctured with minimal patient's discomfort. In addition, this technique was tested in several ex-vivo model catheters and found to be safe and effective. Our technique can be used for non-deflating balloon puncture in female patients in an outpatient setting without anesthesia.

## Introduction

Foley catheters are widely used for urinary drainage in several cases of hospitalized patients. Catheter-related problems and complications are commonly occurring, especially in long-term urethral catheterized patients. These complications include urinary tract infection, bladder pain and patient discomfort, bleeding, catheter leakage and blockage, and rarely catheter retention [1]. Amongst other causes, a non-deflating balloon is the main reason for the foley catheter removal failure that creates an emergency urological problem [2]. Several maneuvers and strategies have been proposed in order to achieve balloon deflation or rupture, which include cutting off the inflation valve, guidewire passing, chemical disruption and surgical puncture of the balloon [3]. We present a simple and safe technique of mechanical puncture of the catheter balloon in female patients with retained catheter.

## Patient and observation

**Figure 1:**
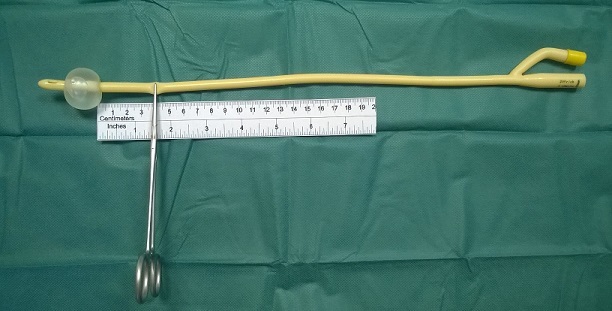
Technique tested in a model latex foley catheter. The catheter is clamped with a Kelly forceps approximately 4 cm to the end of the non-deflated balloon and the insufflation valve is positioned upwards

**Figure 2:**
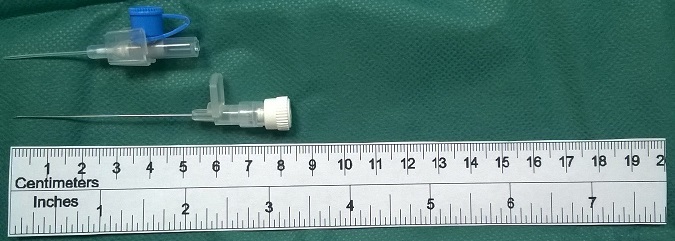
Using the needle of a 20G intravenous catheter set. The needle (5cm long) has sufficient length to reach the balloon

**Figure 3:**
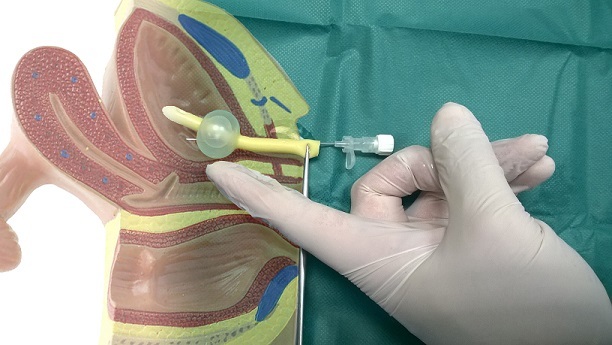
Schematic demonstration of the surgeon's index finger angling the balloon through the vagina while the needle is inserted into the insufflation track and eventually punctures the balloon

A 78-year-old female patient with permanent indwelling catheter due to paraplegia presented in the outpatient's office for catheter replacement. The patient was otherwise healthy, with no concomitant diseases, apart from a history of traumatic spinal cord injury 30 years ago. The latex foley catheter had been replaced 30 days ago and had a normal appearance. Since it was not possible to deflate the balloon initially, even after cutting off the balloon valve, this new technique was performed: The catheter was clamped and preserved with a Kelly forceps proximal to the external urethral orifice omitting the insufflation track Figure 1. No balloon deflation was achieved with this maneuver and thus we proceeded to the next step. A needle of a 20G intravenous catheter set (blue DISPOFLON^®^ I.V. Catheter) was removed from the cannula Figure 2. The detached foley catheter with the forceps was positioned in such a way that the insufflation track was above the clamped urine tract of the catheter. Then, the needle was gently inserted through the insufflation track at approximately 3cm (the needle is 5cm long). A slight upwards pressure was applied to the with the surgeons left index finger which was inserted into the vagina, in order to angulate the distal end of the catheter Figure 3. Finally, the non-deflated balloon was punctured by the needle after completing its insertion and the catheter could be removed. The patient required no anesthesia of any type and the whole procedure was fast and well tolerated in the outpatient's office. The same technique was used in several ex-vivo models including latex foley catheters and silicone catheters in different sizes, effectively.

## Discussion

Following simple maneuvers for catheter removal that have failed, several invasive techniques, even though minimal, are required for needle puncture which is either transvaginal, transabdominal or transrectal [4]. Using these techniques there is an increased risk of tissue injury and complications rate, especially in patients under anticoagulation therapy or problematic cooperation. The transurethral method is effective in women. Still, can result in minor urethral injuries or patient discomfort [5]. Cystoscopic puncture has also been proposed but requires special equipment and it is time-consuming [6]. In our case, as well as in several ex-vivo catheter models, this technique was effective. Our technique exploits the female's urethral anatomy: given that the average length of the female urethra is 3-4cm long [7] and the catheter is cut off to that point, using a 5cm-long needle is sufficient. In addition, the needle is inserted into an already existing passage, which is the insufflation track of the foley catheter, and there is no need for further tissue passages. Our proposed technique ensures minimum pain and very low likelihood of injury. On the other hand, it is limited to female patients and cannot be performed in suprapubic non-deflating catheters, because there is a need of manual angulation of the balloon to be punctured by the needle. For maximal safety, we recommend that the bladder is filled with at least 120cc of water before clamping the catheter, preventing thus, any potential bladder injury from the needle.

## Conclusion

Non-deflating balloon puncture in female patients can be effectively and safely performed using this simple technique in an outpatients' setting without anesthesia.
